# Dynamic enhancer transcription associates with reprogramming of immune genes during pattern triggered immunity in *Arabidopsis*

**DOI:** 10.1186/s12915-022-01362-8

**Published:** 2022-07-21

**Authors:** Ying Zhang, Meng Tang, Mengling Huang, Jiatao Xie, Jiasen Cheng, Yanping Fu, Daohong Jiang, Xiao Yu, Bo Li

**Affiliations:** 1 State Key Laboratory of Agricultural Microbiology, Huazhong Agricultural University, Wuhan, 430070 Hubei China; 2 Hubei Key Lab of Plant Pathology, College of Plant Science and Technology, Huazhong Agricultural University, Wuhan, 430070 Hubei China; 3 Hubei Hongshan Laboratory, Wuhan, 430070 Hubei China

**Keywords:** Plant innate immunity, Transcriptional regulation, Cis-regulatory elements, Enhancers, Enhancer RNAs

## Abstract

**Background:**

Enhancers are cis-regulatory elements present in eukaryote genomes, which constitute indispensable determinants of gene regulation by governing the spatiotemporal and quantitative expression dynamics of target genes, and are involved in multiple life processes, for instance during development and disease states. The importance of enhancer activity has additionally been highlighted for immune responses in animals and plants; however, the dynamics of enhancer activities and molecular functions in plant innate immunity are largely unknown. Here, we investigated the involvement of distal enhancers in early innate immunity in *Arabidopsis thaliana*.

**Results:**

A group of putative distal enhancers producing low-abundance transcripts either unidirectionally or bidirectionally are identified. We show that enhancer transcripts are dynamically modulated in plant immunity triggered by microbe-associated molecular patterns and are strongly correlated with open chromatin, low levels of methylated DNA, and increases in RNA polymerase II targeting and acetylated histone marks. Dynamic enhancer transcription is correlated with target early immune gene expression patterns. Cis motifs that are bound by immune-related transcription factors, such as WRKYs and SARD1, are highly enriched within upregulated enhancers. Moreover, a subset of core pattern-induced enhancers are upregulated by multiple patterns from diverse pathogens. The expression dynamics of putative immunity-related enhancers and the importance of WRKY binding motifs for enhancer function were also validated.

**Conclusions:**

Our study demonstrates the general occurrence of enhancer transcription in plants and provides novel information on the distal regulatory landscape during early plant innate immunity, providing new insights into immune gene regulation and ultimately improving the mechanistic understanding of the plant immune system.

**Supplementary Information:**

The online version contains supplementary material available at 10.1186/s12915-022-01362-8.

## Background

Gene expression in eukaryotes is a complex process regulated by multiple mechanisms. The orchestration of regulatory protein binding to promoters, enhancers, and other cis-regulatory elements facilitates rapid signal-dependent expression changes. Enhancers are noncoding DNA elements that are bound by transcription factors (TFs) and recruit coactivators and transcriptional machinery to stimulate the transcription of target genes [1]. Active enhancers are generally nucleosome-free to facilitate the binding of TFs to their respective DNA motifs. Enhancers are considered important determinants governing the spatiotemporal and quantitative expression dynamics of target genes. In-depth studies have shown that enhancers participate not only in developmental control but also in multiple immune processes [2]. In addition, many sequence variants of noncoding regions available from the Encyclopedia of DNA Elements (ENCODE) project that are responsible for common human diseases, including cancers, are associated with enhancers of relevant disease cell types [3, 4]. With advancements in molecular biology and computational techniques, enhancers have been mapped in plant genomes based on DNase I-hypersensitive site (DHS) and MNase-hypersensitive (MNase HS) regions in Arabidopsis, rice, maize, and cotton, which has greatly promoted the study of plant enhancers in the past few years [5–8]. Similarly, another exquisite study recently identified distal accessible regions (dACRs) in Arabidopsis seedling leaves based on chromatin accessibility profiled by the assay for transposase-accessible chromatin using sequencing (ATAC-Seq), which is a more efficient and less tissue-consuming method for the mapping of open chromatin regions [9]. The identified dACRs showed evolutionary conservation in dicot species and monocot grass species.

Plants have evolved a multilayered innate immune system to defend against pathogens. The proper temporal and spatial regulation of immune gene expression is crucial for translating immune signals into defense-related proteins and ultimately opposing pathogens [10]. The recognition of conserved microbial structures (microbe-associated molecular patterns, MAMPs) or host-derived damage-associated molecular patterns (DAMPs) by membrane-resident pattern recognition receptors (PRRs) triggers complex downstream responses and reprogramming of immune-related gene transcription, leading to PAMP-triggered immunity (PTI) [11]. One well-characterized example is provided by PRR FLAGELLIN-SENSITIVE2 (FLS2), which has been verified to recognize a 22-amino-acid peptide (flg22) derived from bacterial flagellin [12]. Immune-related genes can be induced or repressed within 1 h upon immune signaling activation [13]. A comprehensive understanding of immune gene transcriptional regulatory networks is crucial for providing innovative strategies for crop protection. Immune gene expression in eukaryotic cells is first regulated at the transcription level, a process affected by the coordination between multiple cis- and trans-acting factors. Previous studies have demonstrated that multiple TF families, including WRKY, MYB, NAC, and ERF, activate the expression of immune genes by targeting their promoters [14, 15]. Additionally, some TFs, such as ARABIDOPSIS SH4-RELATED3 (ASR3), could function as transcription repressors to fine-tune immune gene transcription [16]. In particular, EPCOT3 was proven to be a bona fide enhancer in Arabidopsis. EPCOT3 is bound by WRKY33 and induces the transcription of a pathogen-responsive gene, CYP82C2, which further contributes to 4-hydroxy-indole-3-carbonylnitrile (4OH-ICN) biosynthesis and antibacterial defense [17]. However, the systematic identification of active enhancers engaged in the regulation of immune gene expression in plants has yet to be performed.

Active enhancers are associated with low DNA methylation and high levels of acetylation of lysine 27 of histone H3 (H3K27ac) and monomethylation of lysine 4 of histone 3 (H3K4me1) as well as a low level of trimethylation of lysine 4 of histone 3 (H3K4me3) in animals [18, 19]. Recent studies in Arabidopsis have shown the complex relationship between DNA methylation, chromatin accessibility, and 3D genome architecture [20, 21]. However, no single defined type of histone modification provides an excellent marker of distal enhancer activity in Arabidopsis [22]. The modifications of histones and DNA have been demonstrated to play fundamental roles in regulating immune gene expression [23]. For example, the active demethylase REPRESSOR OF SILENCING 1 (ROS1) was found to mediate transcriptional reprogramming and defense in Arabidopsis [24].

In metazoans, active enhancers are enriched with RNA Pol II and general transcription factors (GTFs) and undergo transcription, producing short noncoding enhancer RNAs (eRNAs) [25–27]. eRNAs are generally not spliced or polyadenylated. Critically, RNA synthesis from enhancers precedes mRNA synthesis from target genes and important chromatin remodeling events [28]. The function of eRNA is not entirely clear, but the model of transcription-conducive nucleoprotein architectures forming at enhancers to stimulate target gene transcription is widely accepted [29]. Accordingly, the expression of eRNA has been used to predict the activity of enhancers and induction of target genes [30, 31]. Indeed, the activation of oncogenes or oncogenic signaling pathways often converges upon enhancer activation and production of eRNAs in human cancers [32]. Contrary to the rapidly increasing knowledge of eRNAs in animals, it remains an enigma whether plant enhancers produce similar transcripts.

Here, we defined a putative distal enhancer library based on intergenic open chromatin in Arabidopsis vegetative tissue and used ribosomal RNA (rRNA) depletion RNA-seq to generate a global profile of active enhancer transcription in response to five different immune-activating elicitors in a short time. Most of the enhancer transcripts that we identified with an average length between 100 and 300 bp may be nonpolyadenylated, similar to the eRNA features described in animals. We integrated data from a variety of other genomic assays to provide a comprehensive overview of distal active enhancers and found that the production of eRNA in the distal enhancer region is strongly correlated with transcription-conducive genomic architectures, including an open chromatin architecture, low DNA methylation, the enrichment of RNA poly II, and acetylated histone marks. The examination of intergenic PTI-induced transcribed enhancers reveals their properties as a binding platform for TFs such as WRKYs and SAR DEFICIENT 1 (SARD1) and their functional importance in the regulation of immune genes as well as their high evolutionary conservation across crucifer and dicot species.

## Results

### Genome-wide mapping of putative PTI-related distal enhancers in *Arabidopsis thaliana*

To screen for PTI-related enhancers in Arabidopsis, we first generated an ATAC-Seq library using leaf tissues from wild-type Col-0. Using the common cutoff method, we identified 4460 peaks localized in intergenic regions, which were defined as putative distal enhancers (Additional file 1: Table S1), and the remaining peaks were located in gene-related regions (Additional file 2: Fig. S1a). Given that chromatin accessibility may vary under different experimental conditions, we compared our enhancers with the enhancers identified in Arabidopsis seedling leaves from two previous studies to refine our distal enhancer library [9, 33]. Based on different sequencing methods, the enhancers from the three different sources were merged separately, and we obtained 4702 ATAC-seq-based enhancers (hereafter referred to as enhancers^A^) and 7515 DNase-seq-based enhancers (hereafter, enhancers^D^) (Additional file 3: Table S2). The average lengths of enhancers^A^ and enhancers^D^ after merging were 283 bp and 349 bp, respectively (Additional file 2: Fig. S1b). Approximately 61.5% of the enhancers obtained from ATAC-seq overlapped with distal DHS regions (Additional file 2: Fig. S1b). Thus, we constructed a putative enhancer library of Arabidopsis in the vegetative growth stage based on distal ACRs and DHSs.

**Fig. 1:**
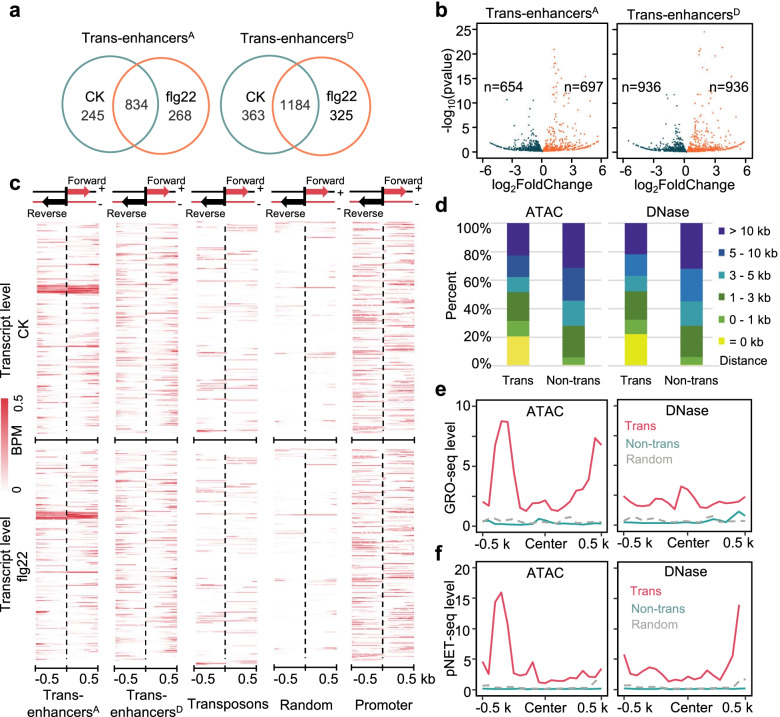
Distal enhancer transcription were responsive to flg22 treatment in *Arabidopsis*. **a** Numbers of transcribed distal enhancer before and after flg22 signaling activation. Trans-enhancers^A^, ATAC-seq-based transcribed enhancers; Trans-enhancers^D^, DNase-seq-based transcribed enhancers. **b** Volcano Plots show differentially expressed transcribed enhancers^A^ and transcribed enhancers^D^ upon flg22 treatment. The numbers of up (*x*-axis > 0, warm orange) and down (*x*-axis < 0, blackish green) transcribed enhancers are shown, respectively. **c** Heatmaps of transcript levels at transcribed enhancers, transposons, promoters (proximal ATAC-seq peaks), and random sequences. Transcript level with and without flg22 treatment were calculated at and around (±0.5 kb) the sequence candidates. The color scales are in BPM for transcript level. **d** Distribution of the mean distance from enhancers to the nearest RNA Pol II peaks. **e**, **f** Distribution of the signals (BPM scale) derived from GRO-seq (**e**) and pNET-seq (**f**) at transcribed enhancers, nontranscribed enhancers, and random sequences. Left and right panels represent ATAC-seq-based enhancers and DNase-seq-based enhancers, respectively

To identify putative distal enhancers involved in PTI responses and immune gene reprogramming, we examined whether enhancer transcripts could be used as active markers. rRNA depletion RNA-seq, which has been proved to be a practical and effective method to identify transcribed enhancers [34], was performed to obtain a global view of eRNA transcripts to assess enhancer expression levels with/without flg22 treatment. We found that active transcription occurred in ∼50% of the genome, corresponding to 336 million reads (>14 Gb), 12% of which were localized in intergenic regions for each sample. We defined potential enhancer transcript regions (see “12” for details) and mapped the rRNA depletion RNA-seq reads. The average expression levels of enhancer transcript regions were calculated as transcripts per million (TPM) values. These enhancers with detectable expression in our analysis were defined as transcribed enhancers, which comprised approximately 25% of total putative distal enhancers. A total of 1079 enhancers^A^ and 1547 enhancers^D^ were expressed without flg22 treatment, while 1102 enhancers^A^ and 1509 enhancers^D^ were expressed 1 h post-flg22 stimulation (Fig. 1a, Additional file 4: Table S3). The transcribed enhancers displayed higher chromatin accessibility than the nontranscribed enhancers, promoters, and random sequences (Additional file 2: Fig. S1c). Then, we calculated the flg22-induced expression differences in individual enhancer transcript regions and identified 697 or 936 upregulated transcribed enhancers from the two libraries (Fig. 1b). These data indicate that a portion of enhancers are actively transcribed and regulated by flg22-mediated immune signaling.

### Global characterization of *Arabidopsis* enhancer transcripts

To investigate the characteristics of enhancer transcripts in Arabidopsis, a sliding window approach was used to identify candidate intergenic regions enriched for eRNA expression. We identified 454 and 543 consecutive transcripts as intact enhancer eRNAs at 0 and 1 h, respectively (Additional file 2: Fig. S1d). The mean length of the identified eRNAs was approximately 200 bp. Only a small portion of the intergenic transcripts and eRNAs could be found in the poly(A) RNA-seq datasets (Additional file 2: Fig. S1d), which suggested that most of the transcribed enhancers were nonpolyadenylated. To study the directionality of Arabidopsis enhancer transcription, we analyzed the distribution of RNA-seq reads on both sides of transcribed enhancers. The strand-specific RNA-seq data obtained in this study provided potentially valuable information for mapping sense and antisense transcripts. Heatmap analysis showed that the expression levels of transcribed enhancers were higher than those of transposons (TEs) and random sequences but lower than those of promoters (Fig. 1c). Approximately 65% of the transcribed enhancers showed bidirectional transcription characteristics, while others were only detected on one strand. These results also showed that flg22 treatment influences the eRNA transcription level of a subset of distal enhancer regions.

To further confirm the authenticity of the enhancer transcription events that we detected, we also cited some published data for analysis [35, 36]. Although most of the identified distal enhancers were located less than 5 kb from the nearest putative transcription start sites (TSSs), the expressed enhancers were closer to the RNA Pol II peaks than the nontranscribed enhancers (Fig. 1d, Additional file 2: Fig. S1e). In particular, approximately 20% of the transcribed enhancers overlapped with RNA Pol II binding peaks, which was not the case for nontranscribed enhancers. Furthermore, the transcription signals derived from GRO-seq and plant native elongating transcript sequencing (pNET-seq) were significantly stronger around transcribed enhancers than around nontranscribed enhancers and random sequences (Fig. 1e, f). The pausing of RNA Pol II and production of nascent RNAs around enhancers further provided direct and reliable measurements of transcribed enhancer activity. Additionally, four reported jasmonate-related enhancers [37] showed open chromatin and increased transcription in response to flg22 (Additional file 2: Fig. S1f). Taken together, our data identified transcribed enhancers, which were further verified by multiple lines of evidence.

### Global characterization of transcribed enhancers in *Arabidopsis*

**Fig. 2:**
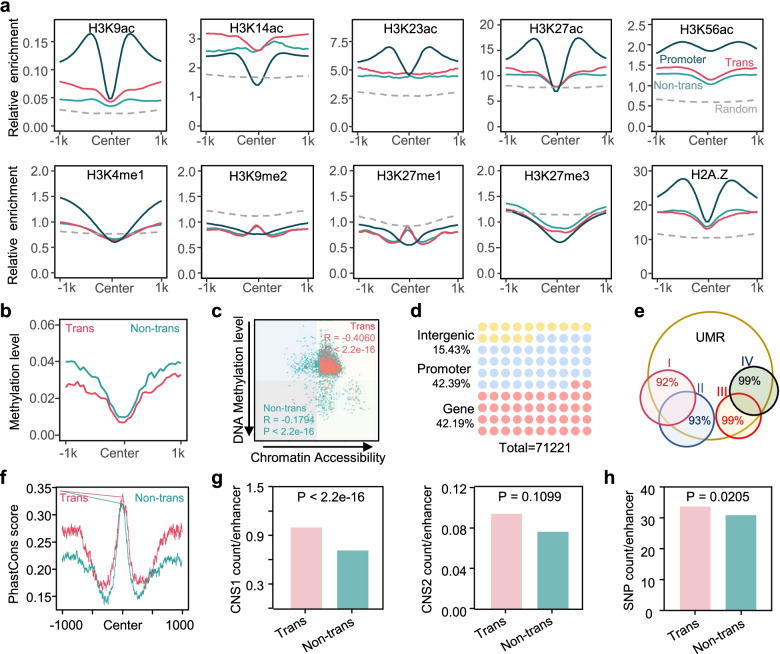
Transcribed enhancers represent a more active and conserved class of distal *cis*-regulatory elements. **a** Distribution of different histone modifications and the histone variant H2A.Z at transcribed enhancers, nontranscribed enhancers, promoter (proximal ATAC-seq peaks), and random sequences. **b** DNA methylation level (mC, weighted average) profiles around transcribed and nontranscribed enhancers. **c** Correlation between DNA methylation level (mC, weighted average) and chromatin accessibility of all transcribed enhancers is represented graphically by a scatterplot. **d** Distribution of the unmethylated regions (UMRs) in *Arabidopsis* genomic regions. The total number of UMRs and genome proportion of each part are shown. **e** Overlap of the total length of enhancers relative to UMRs. The percentage of enhancers overlapping with the UMRs was listed. I, transcribed enhancers^A^; II, nontranscribed enhancers^A^; III, transcribed enhancers^D^; IV, non-transcribed enhancers^D^. **f** Conversation analysis of transcribed and non-transcribed enhancers by PhastCons. **g**, **h** Mean count of conserved noncoding sequences (CNSs) (**g**) and single-nucleotide polymorphisms (SNPs) (**h**) per one hundred transcribed or non-transcribed enhancers. Significant differences among groups were analyzed using the one-tailed Student’s *t* test

The observation of increased chromatin accessibility at transcribed enhancers prompted us to evaluate other chromatin and epigenetic modification characteristics. We first investigated the relative enrichment of a list of histone modifications and the histone variant H2A.Z at enhancers based on public datasets were generated from Arabidopsis vegetative tissues [9, 38–41]. The H3K9ac, H3K23ac, H3K27ac, and H3K56ac levels of transcribed enhancers were lower than those of promoters but higher than those of nontranscribed enhancers and random sequences (Fig. 2a). Among these marks, H3K9ac and H3K27ac are associated with both promoters and enhancers in metazoans and are conserved marks allowing the prediction of proximal DHSs in Arabidopsis [42]. Interestingly, transcribed enhancers were enriched with H3K14ac, which showed even higher levels than in promoter regions (Fig. 2a). In contrast, transcribed enhancers were less associated with the inactive chromatin-associated modification H3K27me3 than nontranscribed enhancers and random enhancers (Fig. 2a). H3K4me1 is specifically enriched at enhancers in metazoans [18] but shows low levels in both transcribed and nontranscribed enhancers in Arabidopsis. H3K9me2 and H3K27me1 levels in the central region of enhancers were higher than those in the flanking sequences and promoters but lower than those in random sequences. In addition, a slightly reduced level of H2A.Z was related to an increase in enhancer transcription activity. Further insight into chromatin characteristics was obtained by computing chromatin state predictions for Arabidopsis intergenic regions based on the relative enrichment levels of these ten histone modification marks by using ChromHMM [43]. The results showed that enhancers were highly related to a silenced state, while promoters were more closely related to a highly active state (Additional file 2: Fig. S2a). Importantly, compared to nontranscribed enhancers, the transcribed enhancer-associated chromatin state was more active.

Recent studies in plants have shown that open chromatin regions are usually accompanied by low levels of DNA methylation. We detected methylation within 1 kb on both sides of the enhancer midpoints. The DNA methylation level of central enhancer regions was approximately 0.01 (Fig. 2b), which was significantly lower than the average methylation level of the whole genome of Arabidopsis [44]. DNA methylation around transcribed enhancers was lower and was more negatively correlated with chromatin accessibility than that around nontranscribed enhancers (Fig. 2c). The plot of methylation and chromatin accessibility levels across 5 chromosomes showed that enhancers, especially transcribed enhancers, were enriched in euchromatin and depleted in heterochromatin (Additional file 2: Fig. S2b). Large constitutively hypomethylated regions in the genome are usually referred to as DNA methylation valleys (DMVs) or unmethylated regions (UMRs), which have unique chromatin characteristics and may contain functional genes and regulatory elements [20, 45]. Therefore, we identified a total of 71221 UMRs in the Arabidopsis genome, among which 15.43% were located in intergenic regions, 42.39% were located in promoter regions, and 42.19% were located in gene body regions (Fig. 2d). To determine whether the identified UMRs and enhancers represent the same regulatory elements, we analyzed the overlap between enhancers and UMRs. Not surprisingly, approximately 99% of the transcribed enhancers were located in UMRs, while nontranscribed enhancers did not show such significant overlap (Fig. 2d). Thus, these results revealed that transcribed enhancers were more likely to be hypomethylated.

The evaluation of chromatin characteristics related to enhancer transcriptional activity impelled us to explore whether enhancer sequences exhibit uneven evolutionary conservation. Hence, we quantified the sequence conservation of enhancers in Arabidopsis and other cruciferous species based on the PhastCons conservation score [46]. Enhancer regions were shown to be more conserved than their flanking regions, and the conservation score of transcribed enhancers was higher than that of nontranscribed enhancers (Fig. 2f). Furthermore, evolutionarily conserved noncoding sequences (CNSs) of Arabidopsis [46, 47] showed higher enrichment within transcribed enhancers (Fig. 2g), which further suggested that transcribed enhancers are more evolutionarily conserved. Enhancer^A^3732, which presented the highest induction fold among the enhancers whose transcription was upregulated by flg22 treatment, was highly conserved among four related species (Additional file 2: Fig. S2e). In addition, the single-nucleotide polymorphisms (SNPs) significantly correlated with lesion size identified within the Arabidopsis/*Botrytis cinerea* pathosystem by a genome-wide association study (GWAS) [48] were more enriched in transcribed enhancers (Fig. 2e). Previous studies using transgenesis assays focusing human and zebrafish developmental enhancers or human and mouse heart enhancers showed a high degree of functional conservation despite sequence divergence [49]. We mapped read coverage across the Arabidopsis genome based on the data described above (Supplementary Fig. 2d). Taken together, the results showed that the transcribed enhancers displayed more potential features related to conservation in evolution and function in plant disease resistance.

### Immune TF binding sites are enriched within PTI-induced enhancers

**Fig. 3:**
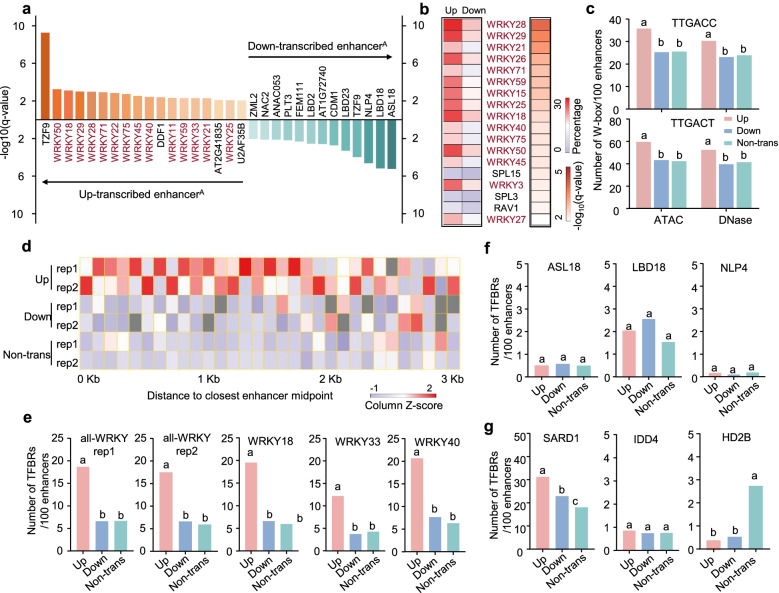
WRKY family transcription factors are enriched in up-transcribed enhancers. **a** Significant enrichment (*P*-value < 0.05) of transcription factors binding motifs in transcribed enhancers^A^ relative to that in non-transcribed enhancers^A^. **b** Significant enrichment (*P*-value < 0.05) of TF binding motifs in upregulated enhancers^A^ relative to that in downregulated enhancers^A^. The percentage of sequences in the target group versus the background group are displayed to the left of the genes. Enrichment *P*-values are listed to the right of the genes as −log10 transformed values. **c**, **e–g** Average number of W-box motif (TTGACC/T) (**c**) and ChIP-seq peaks of transcription factors (**e–g**) in up- or downregulated enhancers, and non-transcribed enhancers. WRKYs binding regions with flg22 treatment for 2 h (**e**); other differentially enriched TFs binding regions (ASL18, mock; LBD18, mock; NLP4, mock) (**f**); and other immune TFs binding regions (HD2B, 30 min after flg22 treatment; IDD4, 1 h after flg22 treatment; SARD1, 24 h after *Pseudomonas syringae* pv*. maculicola* (*P.s.m.*) ES4326 treatment) (**g**). Different letters denote significant differences by the one-way ANOVA test (*P*-value < 0.05). **d** Distribution of distances of peaks for ChIP-seq using the anti-all-WRKY antibody at control condition to the nearest enhancer midpoint

To understand the potential mechanisms of enhancers regulated by flg22, we investigated the enrichment of TF binding motifs in transcribed enhancers relative to that in nontranscribed enhancers. Intriguingly, the majority of the differentially enriched TF motifs (*P*-value < 0.05) in upregulated enhancers were WRKY TF binding sites (Fig. 3a, Additional file 2: Fig. S3a), which are mostly involved in the regulation of plant defense-associated gene expression [50]. In contrast, the differentially enriched TF motifs located in downregulated enhancers were the binding sites of diverse TF families. We further analyzed the motifs enriched in upregulated enhancers using downregulated enhancers as controls. Similarly, the enrichment of WRKY family TF motifs in upregulated enhancers was apparent (Fig. 3b, Additional file 2: Fig. S3b). Additionally, the W-box WRKY TF binding motif, a DNA motif with the core sequence TTGAC(T/C), appeared more frequently in upregulated enhancers (Fig. 3c). Most upregulated enhancer-related TFs exhibited significant upregulation post-flg22 treatment (Additional file 2: Fig. S3c), indicating that these TFs may be implicated in plant innate immunity.

To further confirm the enrichment of WRKY TF binding sites, we integrated the chromatin immunoprecipitation-sequencing (ChIP-seq) results of WRKY TFs obtained with/without flg22 treatment [51]. Relative to downregulated enhancers and nontranscribed enhancers, upregulated enhancers were located closer to the binding peaks identified following ChIP-seq performed with an anti-all-WRKY antibody under normal conditions (Fig. 3d). Upon flg22 treatment, the number of binding peaks for both all-WRKYs and immune-related WRKYs, such as WRKY18, WRKY33, and WRKY40, in upregulated enhancers was almost three times greater than those in other types of enhancers, while no significant difference could be observed between downregulated enhancers and nontranscribed enhancers (Fig. 3e). For instance, Enhancer^A^1671 and Enhancer^A^1286, which are located upstream of the WRKY18 and WRKY40 loci, respectively, showed a dramatic increase in WRKY binding after flg22 treatment (Additional file 2: Fig. S3e). These results suggested that multiple WRKY TFs, inducing the key immune regulators WRKY18, WRKY33, and WRKY40, could preferentially bind to upregulated enhancers, and their binding activity was further promoted by flg22-mediated immune signaling.

We continued to analyze the binding of other differentially enriched TFs to up- and downregulated enhancers. Surprisingly, we found a very low number of binding regions for ASYMMETRIC LEAVES2-LIKE 18 (ASL18), LOB DOMAIN-CONTAINING PROTEIN 18 (LBD18), (NODULE INCEPTION)-LIKE PROTEIN 4 (NLP4), TANDEM ZINC FINGER PROTEIN 9 (TZF9), BASIC HELIX LOOP HELIX PROTEIN (bHLH64), and AT2G41835 TFs in the identified enhancers, and there was no significant difference between those located in up- or downregulated enhancers (Fig. 3f, Additional file 2: Fig. S3d). The ChIP-seq results obtained for HISTONE DEACETYLASE 2B (HD2B), INDETERMINATE-DOMAIN 4 (IDD4) and SARD1 during immune response progression were also included in the analysis [38, 52, 53]. Unlike HD2B and IDD4, the binding peaks of SARD1, an activator of plant immunity that promotes the production of SA and the activation of defense gene expression, were significantly enriched in upregulated enhancers relative to downregulated and nontranscribed enhancers (Fig. 3g). This indicated that upregulated enhancers may recruit specific WRKYs and other immune-related TFs to activate their own transcription and subsequently regulate target immune gene expression.

#### Interactome of enhancers and immune genes with PTI-induced transcription

**Fig. 4:**
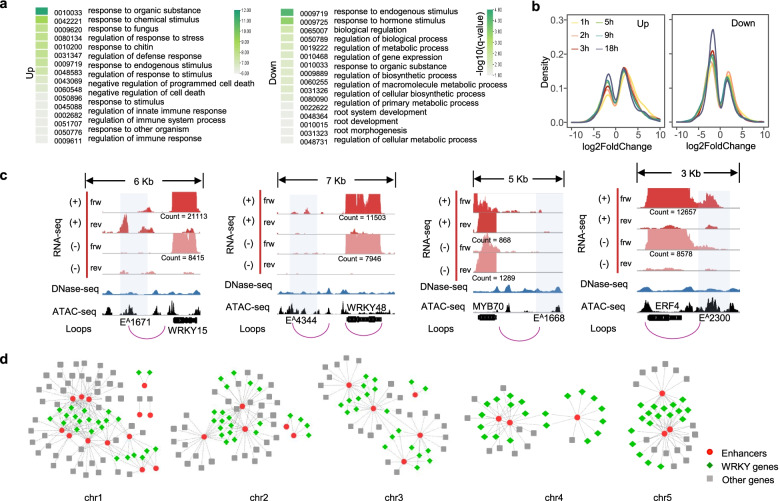
Induction of immune responsive genes are related with the upregulated transcribed enhancers. **a** Enrichment of the closest genes of upregulated enhancers (left panel) and of downregulated enhancers (right panel) with GO terms. **b** Frequency distribution of difference in gene expression, which were from the closest genes of upregulated (left panel) and of downregulated enhancers (right panel), post flg22 treatment compared with mock water treatment. **c** Representative example of immune genes interacted with upregulated enhancers in the loops. Pale blue bars indicate eRNA transcription regions. Plus and minus signs indicate *Arabidopsis* seedlings treated with flg22 and those that were mock-treated, respectively. The raw read counts of each gene are shown. Frw, forward strand; rev, reverse strand. **d** Integrated gene regulatory networks (iGRNs) among upregulated enhancers^A^, WRKY transcription factors and regulatory genes

To dissect the regulatory network between immune-related enhancers and gene expression, we chose different methods to construct the comprehensiveness and accuracy of the interactome of enhancers and genes showing PTI-induced transcription. We first connected enhancers to genes by choosing the closest annotated protein-coding gene to each enhancer (Additional file 5: Table S4). Gene Ontology analysis of the target genes of upregulated enhancers showed that they are mainly linked to the responses to organic substances, stimuli and stresses, regulation of defense responses, innate immune system, and negative regulation of cell death (Fig. 4a). In addition, downregulated enhancer-targeted genes were involved in the response to a hormone stimulus, regulation of biological processes, biosynthetic processes, and metabolic processes (Fig. 4a). To obtain the informative features of target gene transcription dynamics, we referenced time-series RNA-seq data obtained from Arabidopsis leaves in response to flg22 treatment [54], and the expression dynamics of target genes were illustrated in a density map. The closest genes to upregulated enhancers showed significant upregulation in the early stage (1, 2, 3, and 5 h) and presented gradually decreased expression at the middle and late stages (9 and 18 h), while more downregulated enhancer-targeted genes showed decreased expression at all time points (Fig. 4b). The functional analysis and gene expression results indicated that upregulated enhancers play important as-yet-undiscovered roles in the regulation of immune gene expression.

To facilitate gene regulation, distal enhancer elements interact physically with promoter elements via chromatin looping at the TSSs of their target genes [55]. We further studied the spatial relationship between enhancers and target gene expression using statistically significant INT-Hi-C (combining the isolation of nuclei tagged in specific cell types and Hi-C) interaction data with a 2-kb resolution from Arabidopsis leaves [56]. The results showed that enhancers^A^ and enhancers^D^ interacted with 2069 and 3033 genes, respectively, and there were more interaction pairs between transcribed enhancers and genes (Additional file 2: Fig. S4a). Upregulated enhancers were associated with more flg22-treated upregulated genes than downregulated enhancers at 1 h. Some of the genes associated with upregulated enhancers have been proven to play important roles in plant immunity, including important immune genes encoding *WRKY15*, *WRKY48*, *MYB70*, and *ETHYLENE RESPONSIVE ELEMENT BINDING FACTOR 4* (*ERF4)* TFs; immune signaling-related genes such as *MITOGEN-ACTIVATED PROTEIN KINASE KINASE KINASE* 2 (*MAPKKK2*), *JASMONATE-ZIM DOMAIN 9 (JAZ9)*, *AT3G46710* (an NLR gene); and secondary metabolism-related genes such as *CYP71B22* and *CYP71B23* (Fig. 4b, Supplementary Fig. 4b). We also found that some de novo-upregulated enhancers interacted with the target upregulated genes at 0 h, but no enhancer transcripts were detected at this time point, suggesting that the transcription of these enhancers may be induced in a signal-dependent manner. Taken together, the results of the identification of enhancer-gene interactional loops provide an overview of immune gene regulation at the 3D genome level.

Considering the significant binding of WRKYs to upregulated enhancers, we constructed potential integrated gene regulatory networks (iGRNs) based on the assessment of the coexpression intensity between upregulated enhancers carrying W-box motifs with WRKY genes and other regulatory genes to enhance the global understanding of upregulatory interactions. In total, the iGRNs covered 58 enhancers and 252 target genes (954 interactions) (Fig. 4c, Additional file 2: Fig. S4c). Among these sequences, 24 WRKY genes that may play a regulatory role were identified and specifically labeled. In the iGRNs, most enhancers are associated with more than five genes. Enhancer^D^3701, on chromosome 3, showed the highest frequency of gene association. There were 32 genes coexpressed with this enhancer, including 14 WRKYs. Similarly, some genes were associated with multiple enhancers. Based on the networks, the enhancer-TF gene connections were further assessed. Thus, we preliminarily established a causal link between flg22-induced enhancer activation and corresponding immune gene activity on a genome-wide scale based on the perspective of the nearest neighbor strategy, physical interaction, and coexpression.

#### Comparison of different immune elicitors regulated enhancers

**Fig. 5:**
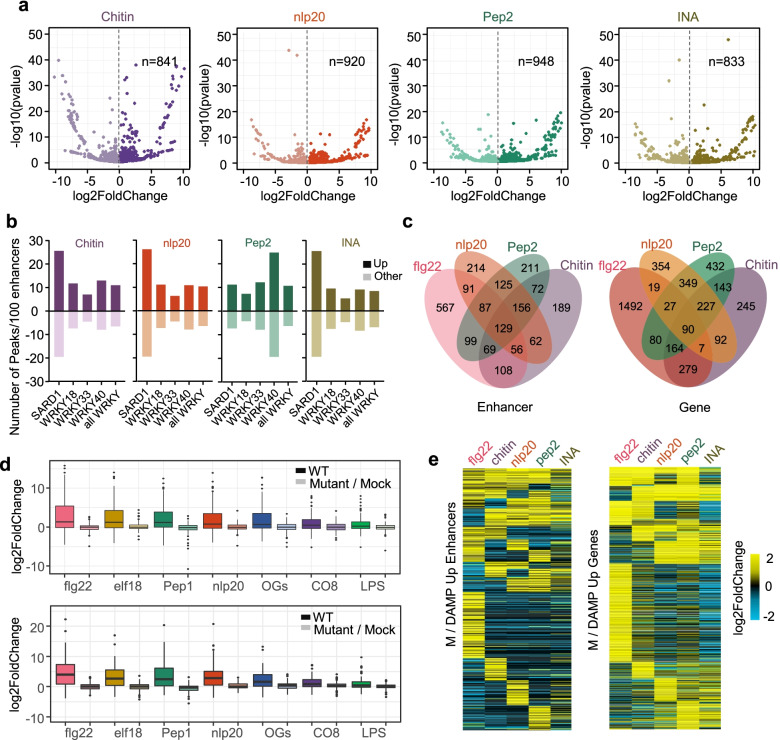
Regulated transcribed enhancers show common characteristic and specificity during multiple PTI signaling. **a** Volcano Plots shown significant differential expressed transcribed enhancers identified upon the treatments of different immune elicitors. The number of upregulated enhancers is shown. **b** Average number of WRKYs and SARD1 binding regions in upregulated enhancers (bright color) and downregulated enhancers (light color). **c** Venn plots showing the overlap number of upregulated enhancers (left panel) and upregulated genes (right panel) that identified upon different patterns treatments. **d** Box plots of sum of fold change in expression (log2 scale) of core pattern-induced enhancers (CPIEs) (upper panel) and core pattern-induced genes (CPIGs) (lower panel), each pattern treatment in long time series (5, 10, 30, 90, and 180 min) in Col-0 wild-type (WT) and cognate receptor mutant. Note that *wak1* mutants are not viable, and thus the OG treatment was paired with a mock water treatment. **e** Heatmaps of fold change in expression (log2 scale) of 4 patterns co-regulating upregulated enhancers (left) and upregulated genes (right) in each elicitor treatments

To further reveal the importance of enhancers in plant innate immunity, we analyzed the enhancers regulated by several other patterns from different source organisms, including chitin (an oligosaccharide fragment released from fungal cell walls), nlp20 (a 20-amino-acid fragment of NECROSIS AND ETHYLENE-INDUCING PEPTIDE 1-LIKE PROTEINS produced by oomycetes and bacterial and fungal microbes), and pep2 (a 23-amino-acid DAMP peptide from Arabidopsis endogenous peptide 2). The analysis also included INA, which is a functional analog of salicylic acid (SA) and can amplify defense response signals as a secondary signal molecule [57, 58]. Using the same workflow and pipeline employed for flg22 data analysis, we identified a similar number of transcribed enhancers during immune responses downstream of these elicitors (Additional file 6: Table S5). In contrast to flg22 elicitation, the numbers of genes and enhancers whose upregulation was induced by these four elicitors were relatively lower. A total of 841, 920, and 948 upregulated enhancers were identified at 1 h after chitin, nlp20, or pep2 treatment, respectively, while more enhancers were detected after flg22 treatment (Fig. 5a). The plant defense hormone INA induced the transcription of 833 enhancers (Fig. 5a, Additional file 7: Table S6). Importantly, the upregulated transcribed enhancers induced by these elicitors similarly showed the enrichment of immune-related SARD1 and WRKY TFs (Fig. 5b), reflecting the convergence between early immune signaling triggered by different patterns.

To further investigate the similarity of transcribed enhancers in different immune events, the commonly upregulated enhancers and upregulated genes induced by all these patterns were screened. There were 129 enhancers that could be upregulated by all tested patterns, which were defined as core pattern-induced enhancers (CPIEs), while different patterns commonly induced the expression of 90 genes that were considered core pattern-induced genes (CPIGs) (Fig. 5c, Additional file 8: Table S7). To demonstrate the core enhancers and genes included in immune signaling pathways triggered by different patterns, we cited gene expression data obtained after stimulation with different several patterns reported in recent publications [38, 59]. The expression of CPIE-linked genes and CPIGs was clearly upregulated after seven different pattern treatments in wild-type plants, but their induction was abolished in the corresponding receptor mutant (Fig. 5d). Gene annotation of the closest CPIE genes revealed that many of them are important plant immune-related genes, such as ERF4 and LecRK-IX.2 (Additional file 8: Table S7). The corresponding CPIEs also showed evolutionary conservation in five cruciferous species (Additional file 2: Fig. S5a). We found that the functions of CPIE-related genes were enriched in the categories of response to stimulus, response to stress, and immune response (Additional file 2: Fig. S5b). The heatmaps showed the expression patterns of upregulated enhancers and genes induced by different patterns (Fig. 5e). Nearly half of the upregulated enhancers were commonly induced by different patterns; however, specific groups of enhancers related to individual patterns could also be found. Notably, the INA-regulated transcribed enhancer set and immune gene set were remarkably different from the set of pattern-upregulated enhancers at 1 h posttreatment (Fig. 5e, Additional file 2: Fig. S5c). Together, these results further indicated that the upregulation of transcribed enhancers is a conserved response downstream of multiple PAMP/DAMP-triggered immune signaling pathways, which may play an important role in regulating immune gene expression.

#### Validation of putative immune-related transcribed enhancers

**Fig. 6:**
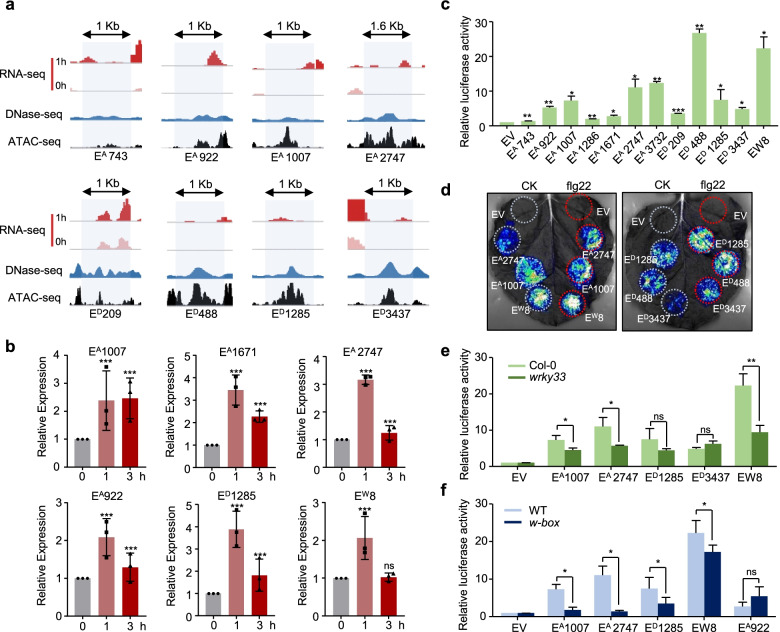
Validation of the expression dynamic and activation of predicted PTI upregulated enhancers. **a** Genome browser view of candidate transcribed enhancers. Pale blue bars indicate eRNA transcription regions. **b** eRNA induction level of candidate transcribed enhancers upon flg22 treatment was verified by RT-qPCR. Ten-day-old seedlings were treated with 100 nM flg22 for 1 and 3 h. The data are shown as mean ± SD from three independent repeats. Different letters indicate significant differences by the one-way ANOVA test (*P* < 0.05). **c** The activation in transcriptional regulation of candidate transcribed enhancers in transient expression assays. *Arabidopsis* protoplasts were infiltrated with constructs of *pGL3B_Enhancer-mini35s::LUC*. The *y*-axis represents the fold enrichment of luciferase signals of each construct compared to the control construct containing the mini 35S promoter. **d** The flg22 treatment increased activity of candidate enhancers. *N. benthamiana* leaf transiently transformed using *Agrobacteria* bacterial containing different constructs. Color scale represents the luminescent signal intensity measured by cps (counts per second). **e** WRKY33 is required for the activity of candidate PTI enhancers. Transient expression assays showing the function in promoting reporter gene expression of candidate enhancers in WT and *wrky33* mutant. **f** The activity of candidate enhancer rely one W-box binding motif. Relative function of wild-type form enhancer and cognate *w-box* mutants were monitored in *Arabidopsis* protoplasts. Data are presented in **c, e, f** as the mean with standard error form six independent biological replicates. The *P*-values were based on a one-tailed Student’s *t* test (*P < 0.05, **P < 0.01; ns, no significance)

To verify the transcription and activation of these candidate transcribed enhancers, we chose representative upregulated enhancers with different characteristics for further testing. In brief, these candidate transcribed enhancers were highly chromatin accessible and were upregulated by one or multiple PAMPs (Fig. 6a, Additional file 2: Fig. S2 and S6a). E^A^1671 was one of the CPIEs whose transcription could be induced by all tested patterns; on the other hand, enhancer Ew8 showed flg22-induced transcription and enriched binding by WRKYs but with a relatively low level of chromatin accessibility (Additional file 2: Fig. S6a). To validate eRNA expression levels, we selected six upregulated enhancers and analyzed them by RT-qPCR. The transcript accumulation of all 6 upregulated enhancers was apparently increased at 1 h after flg22 treatment (Fig. 6c). The expression of E^A^1007 was maintained at a high level even at 3 h, but the expression of the other enhancers was restored to the normal level (Fig. 6c). Additionally, the transcription of CPIE E^A^1671 was also upregulated by chitin, Pep2, and NLP20 but not by INA treatment (Supplementary Fig. 6b). The above data indicated that the eRNAs that we identified were in fact transcribed and could be further induced during immune activation.

Then, the candidate enhancer sequences were cloned and fused with a mini 35S promoter followed by the luciferase reporter gene. We performed transient transcription activity assays in Arabidopsis protoplasts, and all enhancers could induce reporter gene expression relative to the empty vector control (Fig. 6c). To further validate the function of the identified upregulated enhancers in PTI, six of the verified enhancers were tested in transcriptional activity assays in tobacco (*Nicotiana tabacum*) leaves under flg22 treatment. All six enhancers could induce LUC gene expression in tobacco, and more importantly, the induction ability was enhanced by flg22 treatment (Fig. 6d). These results proved the feasibility of identifying active enhancers associated with PTI immunity via our integrated method based on eRNA expression differences.

Notably, WRKY binding motifs were highly enriched in the upregulated enhancers; for instance, E^A^1007, E^A^2747, E^D^1285, and E^A^3437 contained more than one W-box motif. To determine whether WRKY TFs are crucial for enhancer transcript upregulation, we tested their activity in both WT and *wrky33* mutant protoplasts. Three of the five tested enhancers showed reduced transcription activity in the *wrky33* mutant compared with the WT (Fig. 6e), suggesting that WRKY33 contributes to the transcriptional upregulation of certain enhancers. Considering that WRKY TFs exhibit a redundant function and that other WRKYs may be involved in regulation in this context, high-order mutants will be employed for testing in the future. To further confirm that WRKYs directly bind these enhancers to promote transcriptional activity, we performed site-directed mutagenesis of the W-boxes of the candidate enhancers (Additional file 2: Fig. S6c). The w-box mutant variants of most enhancers, except for E^A^922, showed greatly compromised induction of LUC gene transcription activity compared to their wild-type form (Fig. 6f). Thus, these results suggest the critical role of WRKY TFs in regulating PTI-related enhancers. Our results support the notion that the induction of immune gene expression requires a complete enhancer-TF-gene regulatory network.

## Discussion

Although plenty of progress has heretofore been made in the identification of plant enhancers, the exploration of enhancer dynamic activities, especially during the process of plant immunity, is largely incomplete. In this study, we generated and extensively integrated a potential distal enhancer library based on intergenic open chromatin in Arabidopsis. eRNAs that are transcribed unidirectionally or bidirectionally from a subgroup of distal enhancers were identified. The high correlation between eRNA levels and enhancer activities allowed us to rapidly identify enhancers that respond to five different pathogen-related elicitors. The identified PTI-induced transcribed enhancers showed a high density of immune TF binding sites and likely play critical roles in the regulation of immune gene expression. Our findings reveal novel strategies whereby enhancers serve as important modulators of immune gene induction.

We developed a library of active distal enhancers for the early induction of PTI based on plant-fungus, plant-bacteria, and plant-oomycete interaction systems and plant damage-associated pattern recognition. The use of INA, a hormone that can amplify defense signals, further enriched our overall understanding of the regulation of plant immune expression. The numbers of enhancers whose upregulation was induced by different elicitors were similar; however, the greatest number of related transcribed enhancers was upregulated by flg22-triggered signals. Previous studies have shown that flg22 can usually induce a relatively wide range of immune-related gene transcription reprogramming [54, 60]. Moreover, upregulated transcribed enhancers display a strong association with the upregulation of important immune genes, especially early response immune genes, indicating that enhancers positively regulate the immune response. Many of these enhancer-immune gene pairs have already formed a loop structure at 0 h. A recent study analyzed Hi-C data from adult mouse uteruses treated with different hormones for 1 h and found that loop structures were not globally altered by the hormone treatments [61], suggesting that the stimulation by exogenous treatment may not have a great influence on the local chromatin interaction state in a short time. In addition, some genes acquired de novo transcribed enhancers upon flg22 treatment. The gene transcriptional landscape of Arabidopsis pattern-triggered immunity showed that the first 3 h mostly constitute a general pattern-triggered response [59]. We also defined the core set of gene enhancers in response to different patterns. They showed a high correlation with genes that have been proven to play an important role in immunity. The corresponding enhancers were highly conserved in Arabidopsis and other cruciferous species. Furthermore, the future characterization of CIPEs and CIPGs with as-yet-uncharacterized functions or unknown roles in immunity may thus reveal additional PTI players and improve our understanding of the plant PTI gene expression regulatory network. Our work reveals the novel concept of enhancer functions serving as a new and conserved layer of the immune gene regulation system in plants.

Enhancer activity is thought to be regulated by chromatin status and TF binding events [62]. We found that pattern-induced enhancers were significantly enriched with the binding motifs of WRKY family members and SARD1. These TFs regulate numerous target genes involved in the response to biotic stresses, innate immunity, and defense metabolism biosynthesis [63]. Interestingly, W-box motifs were previously found to be overrepresented in gene clusters upregulated early after *Pseudomonas syringae* pv. Tomato DC3000 hrpA- infection [64]. A report of cis-element enrichment in the promoters of immune genes induced 10–30 min after pattern treatment further revealed the association with WRKY families [59]. These data support the core function of WRKYs in promoting gene expression during immunity, not only by direct binding to promoters but also through their enrichment in enhancers to further increase immune gene expression. We further verified this hypothesis by testing enhancer activity in the *wrky33* mutant and the mutation putative W-boxes in candidate enhancers.

In addition, some constitutively expressed WRKYs bind to the promoters of flg22-upregulated WRKY genes and are replaced by inducible WRKYs, such as WRKY18, WRKY33, and WRKY40, upon elicitation [51]. A similar trend was found in the WRKY binding motifs of enhancers with flg22-induced transcription. On the other hand, WRKY gene expression itself could be under feedback regulation during the PTI process, as WRKY18 and WRKY40 bound to their own proximal promoters and associated enhancers after flg22 induction. Therefore, we constructed an integrated immune gene regulatory network among enhancers, WRKY TFs and regulatory genes, in which enhancers could act as a hub. Since the network was established based on expression analysis alone, the possibility of false-positive connections cannot be ignored.

The general features and cell type-specific activities of animal eRNAs have been intensively studied. In contrast, the signatures of plant eRNAs that show low expression levels or are prone to degradation cause great difficulties in their analysis. Several previous studies have revealed very few enhancer signals based on GRO-seq and RNA-seq analyses of Arabidopsis and maize intergenic regions [6, 65]. We speculated that it could be the sequencing depth and coverage that limited the analysis, as not only our data but also those of Zhu et al. in 2018 [35] showed different results. In addition, although GRO-seq and pNET-seq are genome-wide methods to study the nascent transcriptome, the application of the two technologies remains in the primary stage in plants, which may cause unnecessary interference that reduce the accuracy of analysis result [42]. Recent findings have suggested that in Arabidopsis, noncoding RNAs are significantly associated with distal DHSs [5, 22], which might represent eRNAs transcribed from enhancers. Most of the enhancer transcripts that we identified with an average length between 100 and 300 bp may be nonpolyadenylated. Similarly, according to recent studies in animals, the majority of enhancer transcripts are comparatively short, nonpolyadenylated, and nonspliced and function in cis; the others are longer, unidirectional, polyadenylated, and spliced and can function in trans [27]. And the definitions of lncRNAs and eRNAs are not mutually exclusive, which has been discussed in many reviews published in recent years [66]. In general, lncRNA has a polyA tail. And our results show that a small portion of eRNAs could be found in the poly(A) RNA-seq data sets. Therefore, more studies are needed to understand the functions of enhancer RNAs of different lengths in plants.

Changes in eRNA signals have been used as a good indicator of enhancer activity in the identification and elucidation of the functional mechanisms of many immune-related enhancers. For example, the activation of ESR1 can globally increase eRNA transcription in breast cancer [67]. A previous study showed that in T cells, one-third of noncoding RNAs are transcribed from superenhancers, indicating their potential roles in regulating the T cell immune response [68]. Enhancer dynamics during flower development were recently studied through the analysis of chromatin accessibility in Arabidopsis [22]*.* We measured dynamic enhancer activities based on enhancer transcriptional outcomes during PTI from a new perspective in plants for the first time. We evaluated the transcription of distal enhancers in the accessible chromatin regions we identified in the intergenic region. Inevitably, it was not possible to detect all the enhancers undergoing transcription using this method because of limitations such as the reduction of chromatin accessibility by the binding of RNA Pol II and other proteins. Further research could be conducted with the aim of detecting such “omissions.”

Multiple genomic features, including the enrichment of RNA Pol II and specific histone modifications, low levels of DNA methylation, and an open chromatin architecture, are distinctive marks of activated enhancers [69]. Specific histone marks are the best indicators of plant enhancers, and their activity status is slowly emerging. Consistent with previous studies, we observed that H2A.Z, which coexists with DNA methylation marks, preferentially associates with promoters but not enhancers [70]. Published results indicate that active plant enhancers are generally associated with H3 and H4 acetylation, while inactive enhancers appear to be associated with H3K27me3 [42]. We found that transcribed enhancers were more likely to be occupied by positive acetylation marks, which have been shown to reduce chromatin compaction and increase transcription both in vitro and in vivo. Our results indicated that the production of eRNAs from distal enhancers is strongly correlated with the enrichment of these features mentioned above and may be a good indicator of active enhancers. It is not difficult to speculate that eRNA production occurs after or concordant with the assembly of active enhancers.

## Conclusions

By performing high-depth rRNA depletion RNA-Seq and investigating genome-wide chromatin accessibility, DNA methylation, and histone modification, we identified thousands of transcripts corresponding to enhancer regions in Arabidopsis and revealed that the formation of a “transcription hub” is the critical feature of active enhancers and that the production of enhancer transcripts is a good marker of enhancer activity in plants. Based on the differential enhancer expression observed upon elicitation, we generated a library of PTI-related active enhancers in Arabidopsis and screened core pattern-induced enhancers. There is a strong correlation between the expression patterns of enhancers and target genes, and many of them play important roles in regulating plant immunity. Importantly, specific immune-related TFs, including WRKYs and SARD1, were observed to be potential partners of enhancers showing immune-induced transcription. From a general perspective, our study elucidates the comprehensive genome-wide landscape of active enhancers during plant immunity and reveals a mechanistic link consisting of a complete enhancer-TF-gene regulatory network connecting immune gene activation with enhancer dynamics, which ultimately allows hosts to launch a rapid and effective immune response. The results and approaches described in this paper could serve as a resource and provide insightful clues for studying enhancer activity and gene regulation in plant immunity.

## Methods

### Plant materials and growth conditions

The *Arabidopsis thaliana* wild-type and mutant used in this study are in the Col-0 background. The *wrky33-2* mutant (GABI_324B11) was kindly provided by Dr. Xiangzong Meng (Shanghai Normal University). Plants were grown in soil (KEKKILA) in a growth room at 21 °C with 45% humidity, and 75 μE m^−2^ s^−1^ light with a 12-h-light/12-h-dark photoperiod. Ten-day-old seedlings were cultured on half-strength Murashige and Skoog (1/2MS) plates containing 1% sucrose and 0.8% agar, and grown under the same condition as above.

### ATAC-seq and data analyses

Protoplasts were isolated from 4-week-old wild-type *Arabidopsis* plants, and then resuspend in WI solution at a density of 1 × 10^6^ /ml. For ATAT-seq, protoplasts were lysed and the nuclei were collected, the pellet was incubated with transposase Tn5 to construct sequencing libraries. The sequencing was performed by BGI Genomics (Shenzhen, China). Raw reads were trimmed using Trimmomatic (v.0.36) [71]. Trimmed reads were aligned to the reference genome using BWA-MEM (v.0.7.15) [72] with default settings. *Arabidopsis* genome (TAIR10) was obtained from Ensembl (http://plants.ensembl.org/). Aligned reads were sorted using SAMtools (v.1.9) [73], and clonal duplicates were removed using Picard (v.2.20.2). Model-based Analysis of ChIP-seq (MACS2) (v.2.1.2) [74] was used to call peaks with the “-keepdup all” function. To find high-quality peaks, the following filtering steps were generally performed: Peaks called with MACS2 were split into 50 bp windows with 25 bp steps; the Tn5 integration frequency in each window was calculated and normalized to the average frequency in the total genome; windows passing the integration frequency cutoff were merged together with 150 bp gaps; small regions with only one window were then filtered with “length > 50 bp”; regions aligning to the mitochondrial or chloroplast genome were also removed; minimum FDR (*q*-value) cutoff for peak detection is 0.01. The sites within peaks with the highest Tn5 integration frequency were defined as summits. To select for high confidence peaks, only peaks overlapping by at least 50% of their lengths between two replicates were kept for further analysis. For distal enhancers, we focused on intergenic peaks whose center was further than 1.5 kb away from the TSS. When “ATAC-seq-based” or “DNase-seq-based” is not specially labeled, the enhancer dataset represents the union of two sets using BEDTools (v.2.29.0) [75]. If there are less than 50% reciprocal overlap between an enhancer^A^ and enhancer^D^, the enhancer^D^ will be retained; otherwise, the enhancer^D^ will be discarded.

### rRNA depletion RNA-seq and data analysis

The 10-day-old seedlings were transferred to a 6-well tissue culture plate with 2 mL H_2_O for overnight recovery. Then the seedlings were pre-treated with 10 μM CHX for 1 h before further elicitation. Five different elicitors were added to different wells of the pre-treated seedlings and incubated for 1 h. A total of six seedlings were harvested for each biological replicate. The final elicitor concentrations were 100 nM for flg22, 500 μg/ml for chitin, 100 nM for nlp20, 100 nM for Pep2, and 500 μM for INA. rRNA depletion RNA-seq was used to determine whether enhancer drives RNA synthesis at enhancers. Ribosomal RNA was depleted using Ribo-Zero kit. Equal amounts of RNA from biological replicates were pooled for RNA-seq stranded-specific library construction. RNA-seq library preparation and sequencing were performed on an Illunima NovaSeq 6000.

RNA-seq reads with low sequencing quality or reads with sequencing adaptors were filtered from the raw data. The resulting clean reads were then aligned to the *Arabidopsis* reference genome (TAIR10) using Hisat2 (v.2.1.0) [76]. Following the alignments, transcriptome quantification was performed by FeatureCounts in the Subread package (v.1.6.5) [77]. For protein-coding genes, the TAIR10 GFF (general feature format) formatted gene model annotation file was downloaded from Ensembl. For enhancers whose length was less than 1 kb, we used the ± 500 bp of the middle loci of enhancer to define the enhancer transcript regions. For enhancers which were longer than 1 kb, we used the original length to define enhancer transcript regions. For complete eRNAs calling, a sliding window method EnrichedRegionMaker module from USEQ [78] was employed. We filtered out those enhancer transcript regions that are overlapped with known coding regions and lncRNAs (with 1 kb extension from both transcription start sites and transcription end sites). We also excluded all blacklist regions, including known miRNAs, piRNAs, tRNAs, snoRNAs, snRNAs, and rRNAs repeats. We mapped RNA-seq data to these protein-coding genes and enhancer transcript regions and calculated the expression level as TPM in each sample. For enhancer transcription directionality analysis, we used a 25-bp window to count the RNA-seq reads on both sides of the sense and antisense strands of enhancers. The threshold for enhancers to be uni- or bidirectional transcription was *P*-value = 0.05. R package DESeq2 was used to perform differential analysis between two time points [79]. The *P*-values were adjusted for multiple hypothesis testing using the Benjamini-Hochberg procedure [80]. Enhancers were considered as differentially transcribed if they showed at least one-fold change. Genes were considered as differentially expressed if they showed at least twofold changes with FDR < 0.05. GO term enrichment in each gene list was identified using agriGO (v.2.0) [81] with the latest GO term annotations. The cutoff for significant enrichment is *P*-value < 0.05. The fold enrichment was calculated based on the frequency of genes annotated to the term compared with their frequency in the genome.

### WGBS data mapping and analyses

For raw WGBS data analysis, paired-end sequencing reads were first trimmed with Trim Galore (v.0.6.4; http://www.bioinformatics.babraham.ac.uk/projects/trim_galore/) for removal of Illumina adapters and low-quality bases (Phred score < 20). The cleaned reads were then aligned to TAIR10 genome using BSMAP (v.2.90) [82] with default settings. Only uniquely mapped reads were retained for further analysis. PCR duplicates were marked by Picard (v.2.20.2). Further processing was accomplished via SAMtools (v.1.9), BAMtools (v.2.4.0) [83], and bamUtil (v.1.0.2; https://github.com/statgen/bamUtil/). Bisulfite conversion rates were calculated using the unmethylated chloroplast genome as a negative control. Overall, bisulfite conversion rate was > 99% in all the samples. To increase sequencing coverage, we merged the data from two biological replicates. Methylation ratio was extracted with BatMeth2 (https://github.com/GuoliangLi-HZAU/BatMeth2/).

### Identification of unmethylated regions

We adopted a similar approach in Crisp et al. [20] to identify unmethylated regions (UMRs). The average methylation level at a 100-bp sliding window (step = 100 bp) was calculated for the whole genome. Briefly, windows were classified as missing data if there was less than 10× coverage, and as unmethylated regions if total percentage of mCG, mCHG, and mCHH were less than 10%. Following window classification, adjacent unmethylated windows were merged. To capture and combine unmethylated regions that were fragmented by a short interval of missing data (low coverage or no sites), any merged unmethylated window regions that were separated by missing data were merged if the resulting merged region consisted of no more than 33% missing data. Regions more than 300 bp were defined as UMRs. We classified UMRs within 1500 bp of the annotated TSS as gene-proximal, and UMRs greater than 1500 bp as gene-distal; however, if a UMR overlapped with both the gene locus and the gene-proximal region, it was hierarchically classified as proximal.

### Phylogenetic analysis and motif enrichment

All phylogenetic species trees were adapted from published Data. We used TIMETREE (www.timetree.org), which synthesizes divergence times to estimate the timescale of Brassicaceae species evolution [48, 84–96]. Sequence alignment plots were generated using mVISTA (http://genome.lbl.gov/vista/mvista/submit.shtml). All the enhancers were adjusted to the same size (500 bp) centered peak midpoint. AME were used to identify differentially enriched motifs between two datasets [97].

### Network generation and visualization

Global coexpression networks were constructed from RNA-seq data derived from both control and flg22-treatment conditions. The Pearson correlation coefficient value (*r*) was first calculated based on the expression profiles for each enhancer-gene pair. The coexpression links in the network were kept if the corresponding *r* values are at or above a threshold. The threshold value was determined according to the scale-free criterion, which is measured by the square of the correlation coefficient (*R*) between log(*P*(*k*)) and log (*k*), where *k* denotes the connectivity of a node, or the number of links of a node to other nodes in a network. *P*(*k*) gives the probability that a selected node has exactly *k* links, which is calculated as the number of the nodes (genes) at a given *k* value divided by the total number of nodes. The *R* value of 0.95 corresponded to *P*-value < 0.001, so that was selected as the threshold value. Genes with *R* values at or above the threshold value were coexpressed. The upregulated enhancers containing W-box and its 1 Mb upstream and downstream genes were retained. We used Cytoscape (http://www.cytoscape.org/) to visualize the resulting network.

### Data visualization and file generation

Bigwig software files of RNA-seq and ATAC-seq data for Integrated Genome Viewer (v.2.3.57) visualization were converted to BigWig files using deepTools (v.3.0.2) [98]. The signal densities for ATAC-seq, DNA methylation, eRNA expression, and histone ChIP-seq are from 1 kb upstream to 1 kb downstream around enhancer midpoints. The heatmap was generated using the TBtools software [99] and R package ComplexHeatmap [100]. If not specified, R (v.3.5.1) was used to compute statistics and generate plots. INT-Hi-C interaction data were visualized in the WashU Epigenome Browser (https://epgg-test.wustl.edu/browser/).

ChIP-seq data and bigwig files were retrieved from GEO dataset GSM3674620 (H3K4me3) [9], GSE51304 (H3K9me2) [41], GSM4455286 (H3K14ac) [101] , GSE51304 (H3K23ac) [41], GSE86498 (H3K23me1) [102], GSM567818 (H3K27me1) [24], GSM3674617 (H3K36me3) [9], and GSM3674619 (H3K56ac) [9], GSE112443 (RNA Pol II) [36]. Peaks for DAP-seq/ampDAP-seq of TFs (ASL18, LBD18, NLP4, TZF9, bHLH64, AT2G41835) were retrieved from Plant Cistrome Database [103]. The GRO-seq and pNET-seq were retrieved from GEO dataset GSE109974 [35].

### RNA isolation, cDNA synthesis, and RT-qPCR

Total RNA was extracted from 10-day-old seedlings by TRNzol reagent and quantified with NanoDrop. cDNA was synthesized from 1 μg total RNA using HisScript II Q RT SuperMix for qPCR (Vazyme). SYBRGreen master mix was used for Quantitative PCR (qPCR) reactions. The expression of each gene was normalized to the expression of *ACTIN2*. Three independent biological replicates were analyzed.

### Cloning and mutagenesis

To generate reporter constructs, the candidate enhancer segments were PCR-amplified from *Arabidopsis* Col-0 genomic DNA, digested with KpnI and HandIII, and ligated into the *pGL3-basic* vector in front of the *Minimal* 35S (*mini35S*) which drives the *Luciferase* (*LUC*) gene expression. The point mutations of indicated W-box variants were generated by PCR-based site-directed mutagenesis. To construct reporter in the binary vectors, the whole expression cassette containing *enhancer-mini35S-LUC-NOS* were released by EcoRI and BamHI and ligated into the *pCAMBIA2300* vector. The above binary constructs were introduced into *Agrobacterium tumefaciens* GV3101 by electroporation. All the recombinant plasmids were confirmed by nucleotide sequencing.

### Reporter assay in Arabidopsis protoplasts

Protoplasts were isolated from 4-week-old wild-type *Arabidopsis* plants at a density of 2 × 10^5^/ml. To determine the enhancer activity, protoplasts transfected with the reporter constructs were collected at 8–10 hpt. *UBQ10-GUS* was co-transfected as an internal transfection control, and the enhancer activity was presented as LUC/GUS ratio. Protoplasts transfected with empty *pGL3-basic* vector were used as reporter controls. Six independent biological replicates were included for each treatment.

### Agroinfiltration of Nicotiana benthamiana leaves and LUC assay

*Nicotiana benthamiana* plants were grown in a greenhouse for 3–4 weeks before the agroinfiltration step. *A. tumefaciens* GV3101 containing the binary vector were cultured at 28 °C in LB liquid medium with 100 μg/ml Kanamycin and 100 μg/ml Gentamicin. Bacteria were harvested by centrifugation at 3000 rpm for 15 min, and the pellet was suspended with buffer containing 10 mM MES (pH 5.7), 10 mM MgCl_2_, and 200 μM acetosyringone at the density of OD600 = 0.2. After 3 h incubation at RT, bacterial solutions were infiltrated into the abaxial side of tobacco leaves with a needleless syringe. flg22 (100 nM) or ddH_2_O treatment for 6 h was performed at 24 h post gene expression. Leaves were then sprayed with 1 mM D-luciferin solution containing 0.01% Triton X-100 and incubated in darkness for 5 min. The bioluminescence of whole leaves was visualized with the Chemiluminescent Imaging System (Tanon 5200) to record the photon emission over a 15-s interval. For each experiment, three independent biological replicates were performed.

## Supplementary Information

**Additional file 1: Table S1**. List of putative distal enhancer peaks based on ATAC-seq.

**Additional file 2: Figure S1**. Genome-wide characterization of enhancers based on chromatin accessibility and transcript level. **Figure S2**. Chromatin characteristics and evolutionary conservation of transcribed enhancers. **Figure S3**. Immune regulators show relative enrichment in flg22 induced up-regulated transcribed enhancers. **Figure S4**. Interactome between flg22 up-regulated transcribed enhancers and immune related genes. **Figure S5**. Potential function of core pattern induced enhancers during immunity. **Figure S6**. Expression and function validation of a core pattern induced enhancer.

**Additional file 3: Table S2**. List of putative distal enhancers in the integrated library.

**Additional file 4: Table S3**. List of transcribed enhancers with/without flg22 treatment.

**Additional file 5: Table S4**. List of genes close to flg22-regulated transcribed enhancer and expression dynamic.

**Additional file 6: Table S5**. Collection of different PAMPs-regulated transcribed enhancers.

**Additional file 7: Table S6**. Collection of different INA-regulated transcribed enhancers.

**Additional file 8: Table S7**. List of CIPEs and neighbor genes.

**Additional file 9: Table S8**. Primers used in this study.

## Data Availability

All data generated or analyzed during this study are included in this published article, its supplementary information files and publicly available repositories. The rRNA depletion RNA-seq and ATAC-seq data were deposited in the Gene Expression Omnibus accession number GSE181721 [104] at the National Center for Biotechnology Information. DNA methylation Whole-Genome Bisulfite Sequencing (WGBS) data and poly(A) RNA-seq files were retrieved from GEO dataset GSE156608 [105].

## Abbreviations

4OH-ICN: 4-Hydroxy-indole-3-carbonylnitrile
ASL18: ASYMMETRIC LEAVES2-LIKE 18
ASR3: ARABIDOPSIS SH4-RELATED3
ATAC-Seq: Transposase-accessible chromatin using sequencing
bHLH64: BASIC HELIX LOOP HELIX PROTEIN
ChIP-seq: Chromatin immunoprecipitation-sequencing
CNS: Conserved noncoding sequence
CPIE: Core pattern-induced enhancer
CPIG: Core pattern-induced gene
dACR: Distal accessible region
DAMP: Damage-associated molecular pattern
DHS: DNase I-hypersensitive site
DMV: DNA methylation valley
ENCODE: Encyclopedia of DNA elements
enhancer^A^: ATAC-seq-based enhancer
enhancer^D^: DNase-seq-based enhancer
ERF4: ETHYLENE RESPONSIVE ELEMENT BINDING FACTOR 4
eRNA: Enhancer RNA
FLS2: FLAGELLIN-SENSITIVE2
GTF: General transcription factor
GWAS: Genome-wide association study
H3K27ac: Acetylation of lysine 27 of histone H3
H3K4me1: Monomethylation of lysine 4 of histone 3
H3K4me3: Trimethylation of lysine 4 of histone 3
HD2B: HISTONE DEACETYLASE 2B
IDD4: INDETERMINATE-DOMAIN 4
iGRN: Integrated gene regulatory network
INT-Hi-C: Combining the isolation of nuclei tagged in specific cell types and Hi-C
JAZ9: JASMONATE-ZIM DOMAIN 9
LBD18: LOB DOMAIN-CONTAINING PROTEIN 18
MAMP: Microbe-associated molecular pattern
MAPKKK2: MITOGEN-ACTIVATED PROTEIN KINASE KINASE KINASE 2
MNase HS: MNase-hypersensitive
NLP4: NODULE INCEPTION)-LIKE PROTEIN 4
pNET-seq: Plant native elongating transcript sequencing
PRR: Pattern recognition receptor
PTI: PAMP-triggered immunity
ROS1: REPRESSOR OF SILENCING 1
rRNA: Ribosomal RNA
SARD1: SAR DEFICIENT 1
SNP: Single-nucleotide polymorphism
TE: Transposon
TF: Transcription factor
TPM: Transcripts per million
TSS: Transcription start site
TZF9: TANDEM ZINC FINGER PROTEIN 9
UMR: Unmethylated region
