# Supplementary material for: Dynamic enhancer transcription associates with reprogramming of immune genes during pattern triggered immunity in Arabidopsis
Source: BMC Biol. 2022 Jul 21;20:165. doi: 10.1186/s12915-022-01362-8 (PMC9301868; doi:10.1186/s12915-022-01362-8)
Supplement: Supplementary file 2 — Additional file 2: Figure S1. Genome-wide characterization of enhancers based on chromatin accessibility and transcript level. Figure S2. Chromatin characteristics and evolutionary conservation of transcribed enhancers. Figure S3. Immune regulators show relative enrichment in flg22 induced up-regulated transcribed enhancers. Figure S4. Interactome between flg22 up-regulated transcribed enhancers and immune related genes. Figure S5. Potential function of core pattern induced enhancers during immunity. Figure S6. Expression and function validation of a core pattern induced enhancer. [file 12915_2022_1362_MOESM2_ESM.pdf]

Figure S1

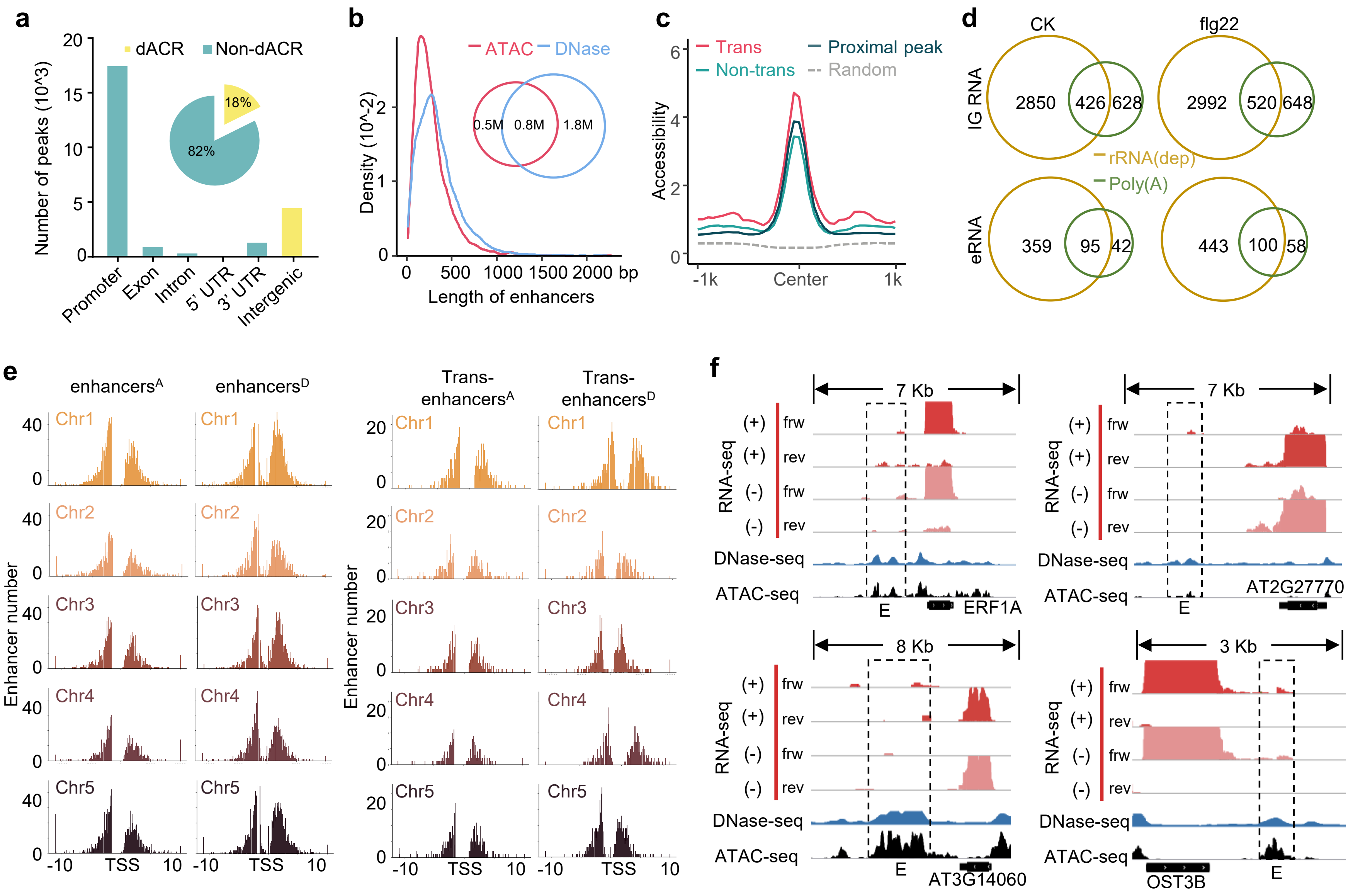

a
Number of peaks (10^3)
dACR
Non-dACR
18%
82%
Promoter
Exon
Intron
5' UTR
3' UTR
Intergenic
b
ATAC
DNase
0.5M
0.8M
1.8M
Density (10^-2)
Length of enhancers
c
Trans
Non-trans
Proximal peak
Random
Accessibility
-1k
Center
1k
d
CK
flg22
IG RNA
eRNA
2850
426
628
2992
520
648
359
95
42
443
100
58
rRNA(dep)
Poly(A)
e
enhancersA
enhancersD
Trans-enhancersA
Trans-enhancersD
Chr1
Chr2
Chr3
Chr4
Chr5
Enhancer number
-10
TSS
10
f
7 Kb
8 Kb
3 Kb
RNA-seq
(+) frw
(+) rev
(-) frw
(-) rev
DNase-seq
ATAC-seq
E
ERF1A
AT2G27770
AT3G14060
OST3B

Figure S2

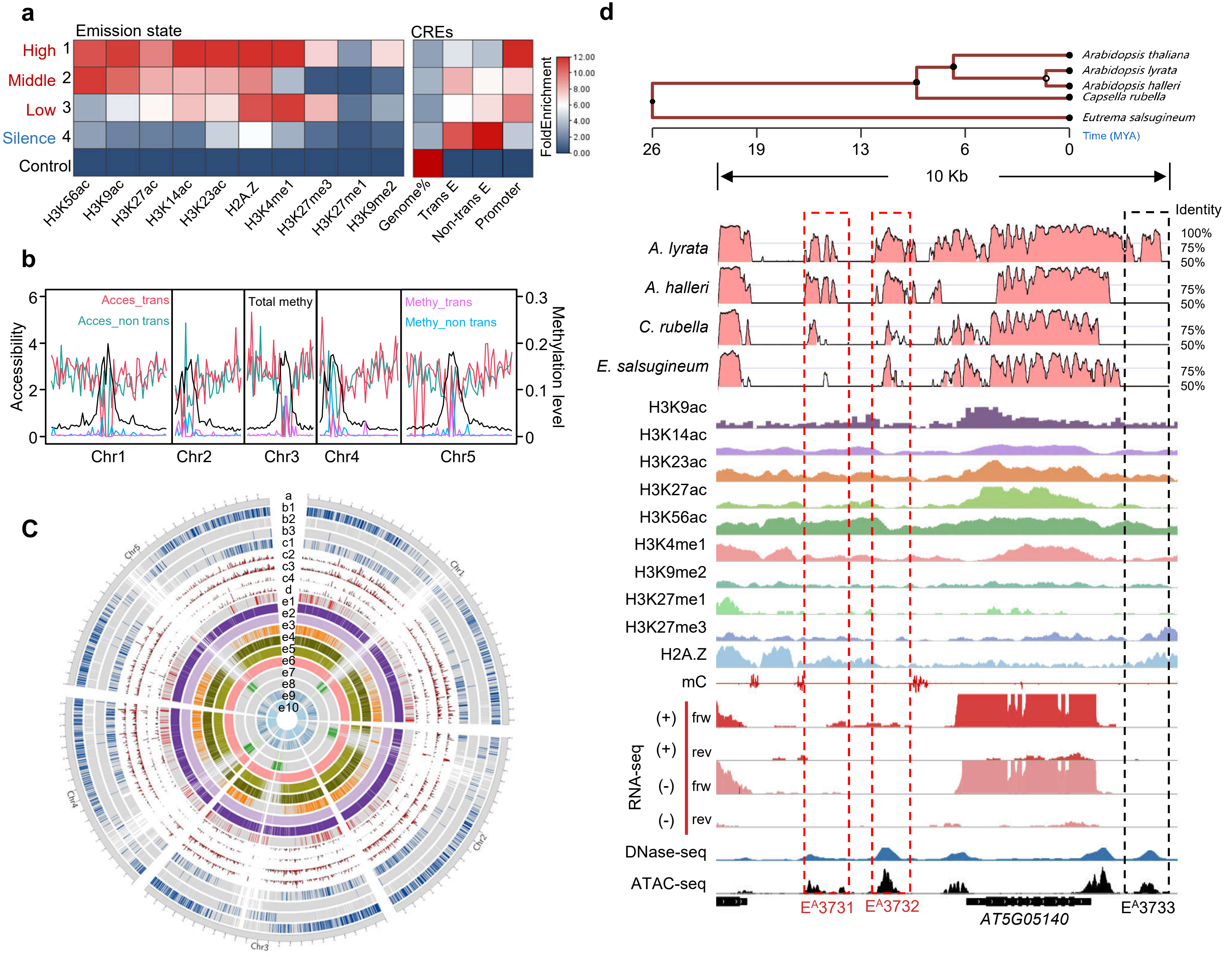
a
Emission state
CREs
High 1
Middle 2
Low 3
Silence 4
Control
H3K56ac
H3K9ac
H3K27ac
H3K14ac
H3K23ac
H2A.Z
H3K4me1
H3K27me3
H3K27me1
H3K9me2
Genome%
Trans E
Non-trans E
Promoter
FoldEnrichment
b
Accessibility
Methylation level
Acces_trans
Acces_non trans
Total methy
Methy_trans
Methy_non trans
Chr1
Chr2
Chr3
Chr4
Chr5
C
a
b1
b2
b3
c1
c2
c3
c4
d
e1
e2
e3
e4
e5
e6
e7
e8
e9
e10
d
Arabidopsis thaliana
Arabidopsis lyrata
Arabidopsis halleri
Capsella rubella
Eutrema salsugineum
Time (MYA)
26
19
13
6
0
10 Kb
Identity
A. lyrata
A. halleri
C. rubella
E. salsugineum
100%
75%
50%
H3K9ac
H3K14ac
H3K23ac
H3K27ac
H3K56ac
H3K4me1
H3K9me2
H3K27me1
H3K27me3
H2A.Z
mC
RNA-seq
(+) frw
(+) rev
(-) frw
(-) rev
DNase-seq
ATAC-seq
E^A3731
E^A3732
AT5G05140
E^A3733

Figure S4

a

		Number of Enhancers	Number of Interactions with Genes	Number of Interacted Genes	Ratio (Interacted Gene /Enhancer)	Interactions with Up Genes*	Ratio (Interactions with Up Genes /Enhancers)
Enhancer^{A}	All	4702	2729	2069	0.44	282	0.06
	Up	697	424	387	0.56	88	0.12
	Down	650	450	408	0.63	19	0.03
	De novo-up	268	128	118	0.44	16	0.06
Enhancer^{D}	All	7515	5200	3733	0.50	463	0.06
	Up	936	642	594	0.63	106	0.11
	Down	936	701	646	0.69	37	0.04
	De novo-up	325	178	169	0.52	18	0.06

Up Genes*: A total of 2001 genes that were up-regulated after flg22 treatment for 1 h.

b

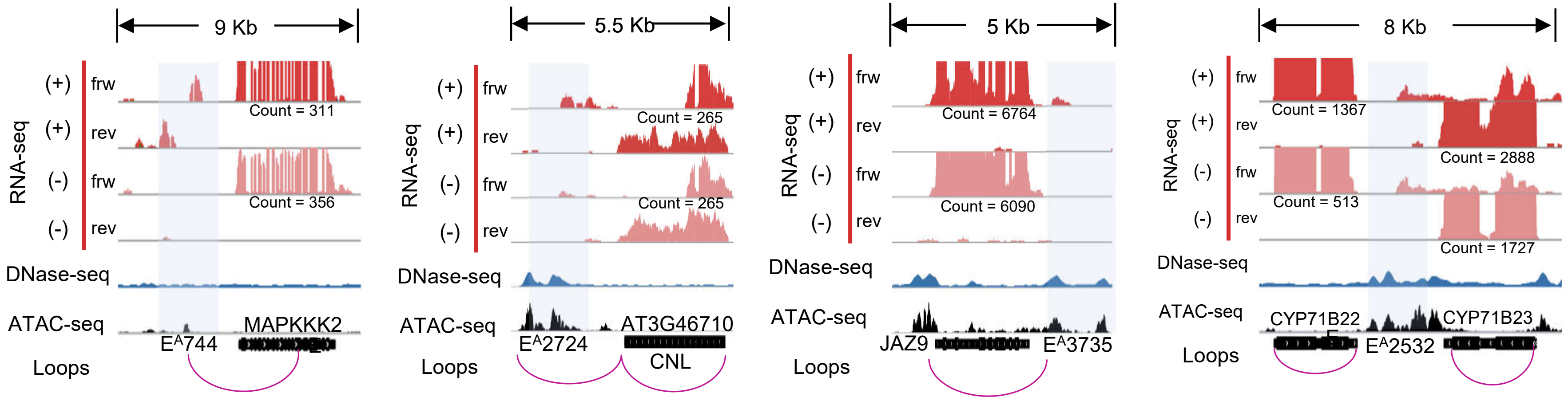

c

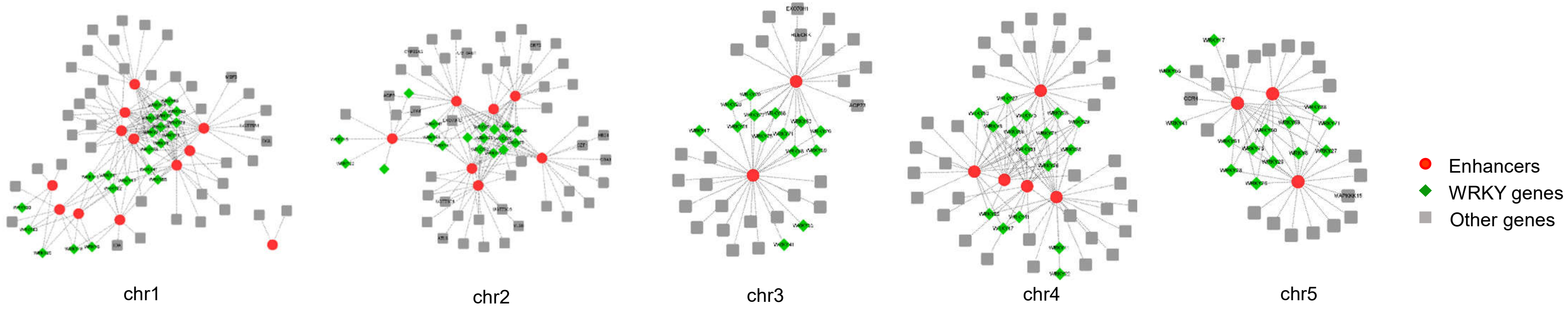

Figure S3

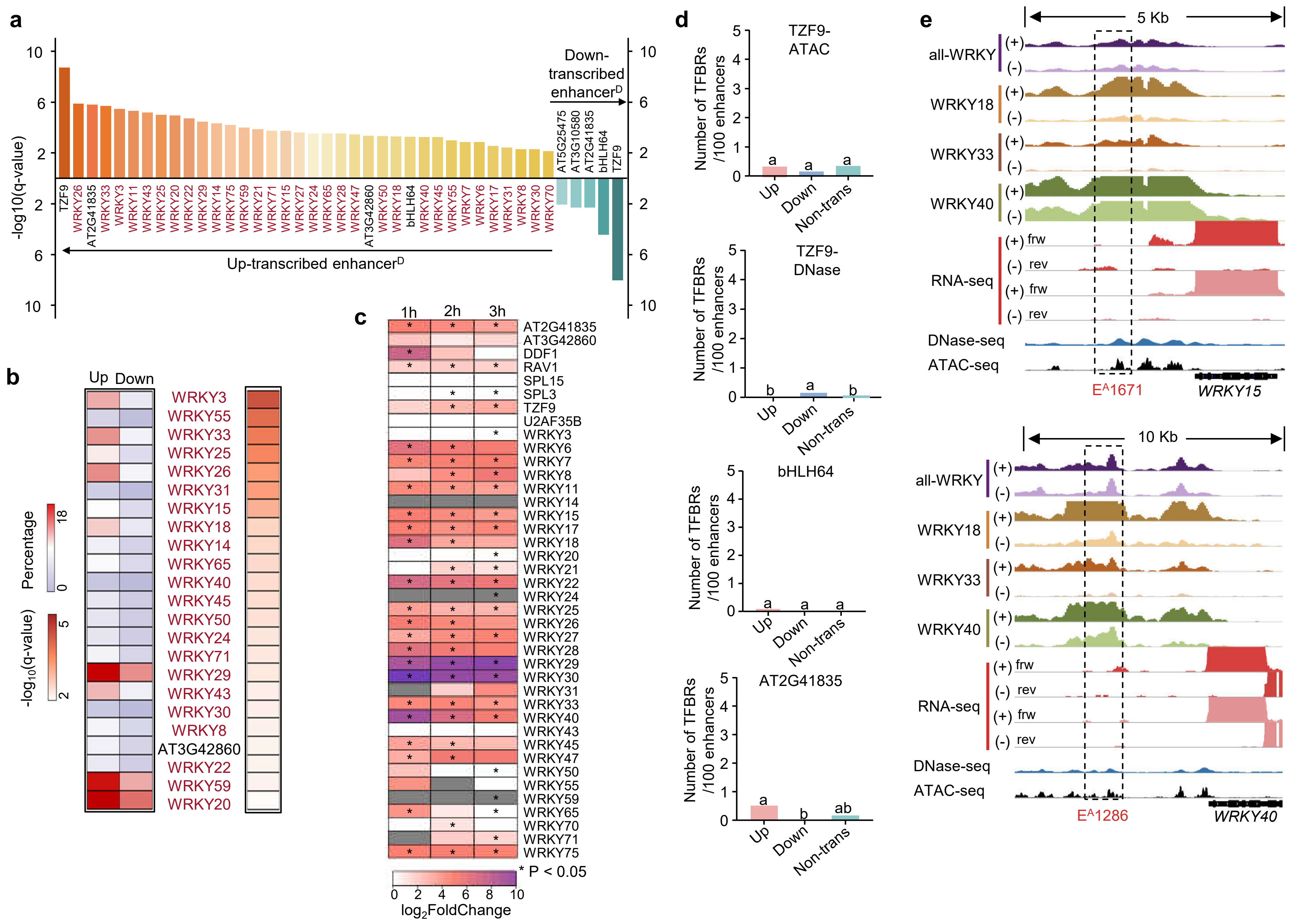
a
-log10(q-value)
Down-transcribed enhancer^D
Up-transcribed enhancer^D
TZF9
WRKY26
AT2G41835
WRKY33
WRKY3
WRKY11
WRKY43
WRKY25
WRKY20
WRKY22
WRKY29
WRKY14
WRKY75
WRKY59
WRKY21
WRKY71
WRKY15
WRKY27
WRKY24
WRKY65
WRKY28
WRKY47
AT3G42860
WRKY50
WRKY18
bHLH64
WRKY40
WRKY45
WRKY55
WRKY7
WRKY6
WRKY17
WRKY31
WRKY8
WRKY30
WRKY70
AT5G25475
AT3G10580
AT2G41835
bHLH64
TZF9
b
Up Down
Percentage
-log$_{10}$(q-value)
WRKY3
WRKY55
WRKY33
WRKY25
WRKY26
WRKY31
WRKY15
WRKY18
WRKY14
WRKY65
WRKY40
WRKY45
WRKY50
WRKY24
WRKY71
WRKY29
WRKY43
WRKY30
WRKY8
AT3G42860
WRKY22
WRKY59
WRKY20
c
1h 2h 3h
AT2G41835
AT3G42860
DDF1
RAV1
SPL15
SPL3
TZF9
U2AF35B
WRKY3
WRKY6
WRKY7
WRKY8
WRKY11
WRKY14
WRKY15
WRKY17
WRKY18
WRKY20
WRKY21
WRKY22
WRKY24
WRKY25
WRKY26
WRKY27
WRKY28
WRKY29
WRKY30
WRKY31
WRKY33
WRKY40
WRKY43
WRKY45
WRKY47
WRKY50
WRKY55
WRKY59
WRKY65
WRKY70
WRKY71
WRKY75
* P < 0.05
log$_2$FoldChange
d
Number of TFBRs /100 enhancers
TZF9-ATAC
TZF9-DNase
bHLH64
AT2G41835
Up
Down
Non-trans
e
5 Kb
10 Kb
all-WRKY
WRKY18
WRKY33
WRKY40
RNA-seq
frw
rev
DNase-seq
ATAC-seq
E^{A}1671
WRKY15
E^{A}1286
WRKY40

Figure S5

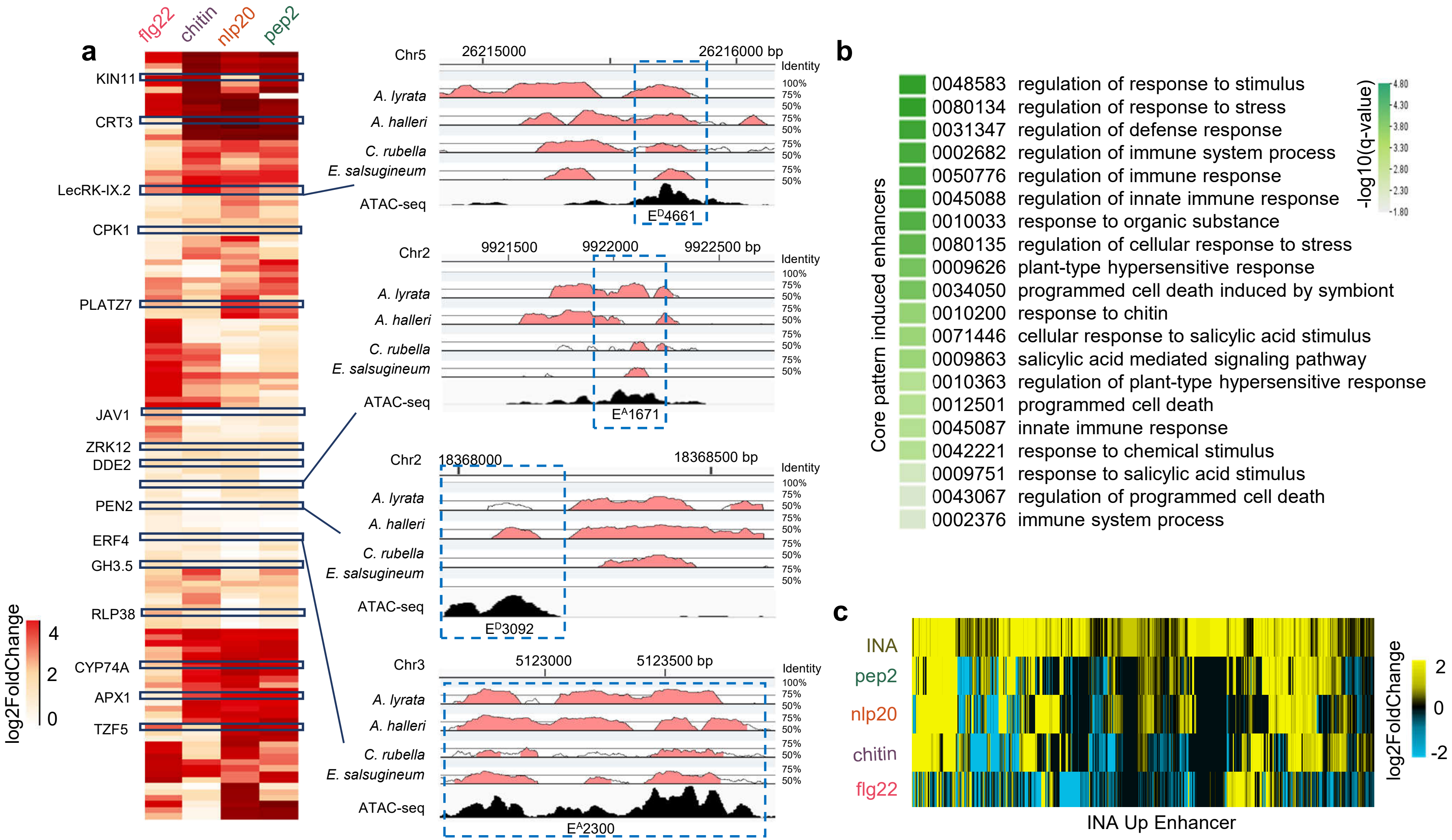
a
flg22
chitin
nlp20
pep2
KIN11
CRT3
LecRK-IX.2
CPK1
PLATZ7
JAV1
ZRK12
DDE2
PEN2
ERF4
GH3.5
RLP38
CYP74A
APX1
TZF5
log2FoldChange
4
2
0
Chr5
26215000
26216000 bp
Identity
A. lyrata
A. halleri
C. rubella
E. salsugineum
ATAC-seq
E^D4661
Chr2
9921500
9922000
9922500 bp
E^A1671
18368000
18368500 bp
E^D3092
Chr3
5123000
5123500 bp
E^A2300
100%
75%
50%
b
Core pattern induced enhancers
0048583 regulation of response to stimulus
0080134 regulation of response to stress
0031347 regulation of defense response
0002682 regulation of immune system process
0050776 regulation of immune response
0045088 regulation of innate immune response
0010033 response to organic substance
0080135 regulation of cellular response to stress
0009626 plant-type hypersensitive response
0034050 programmed cell death induced by symbiont
0010200 response to chitin
0071446 cellular response to salicylic acid stimulus
0009863 salicylic acid mediated signaling pathway
0010363 regulation of plant-type hypersensitive response
0012501 programmed cell death
0045087 innate immune response
0042221 response to chemical stimulus
0009751 response to salicylic acid stimulus
0043067 regulation of programmed cell death
0002376 immune system process
-log10(q-value)
4.80
4.30
3.80
3.30
2.80
2.30
1.80
c
INA
pep2
nlp20
chitin
flg22
INA Up Enhancer
log2FoldChange
2
0
-2

Figure S6

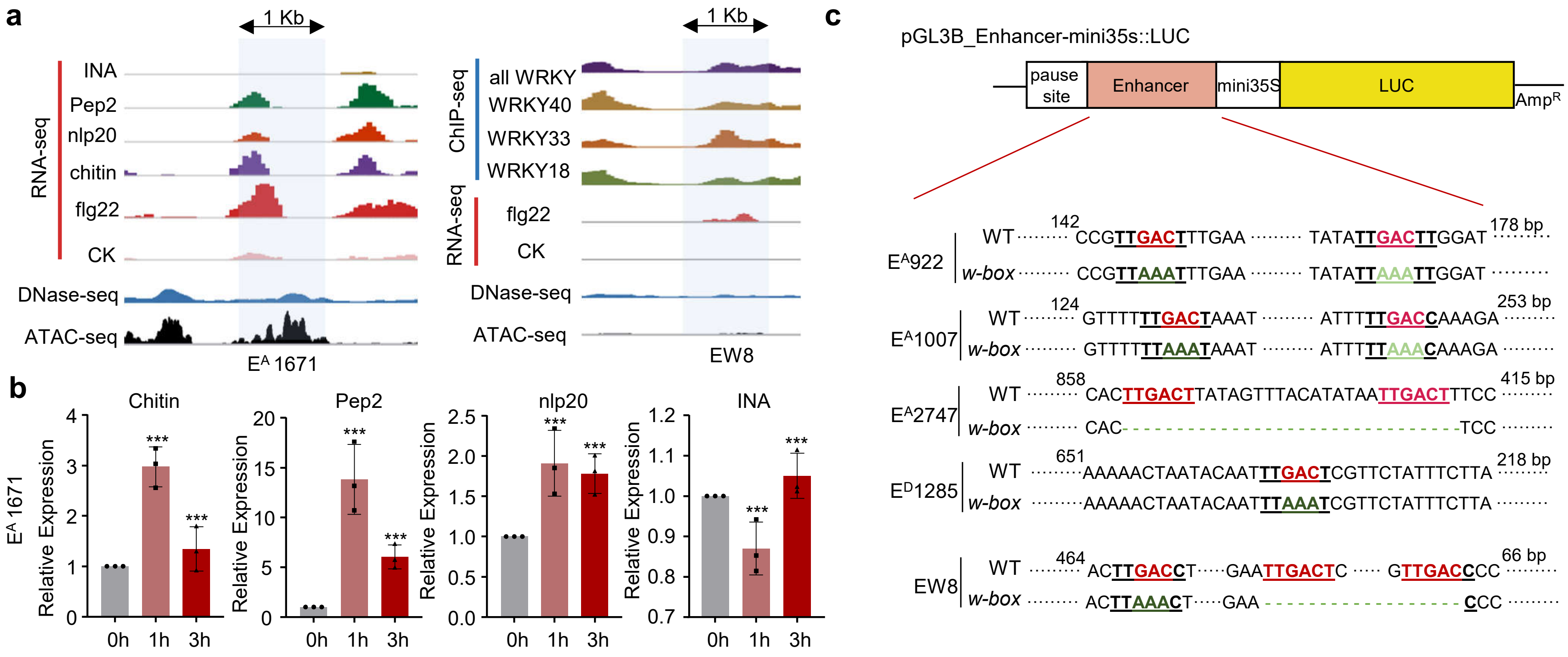
a
1 Kb
INA
Pep2
nlp20
chitin
flg22
CK
RNA-seq
DNase-seq
ATAC-seq
EA 1671
all WRKY
WRKY40
WRKY33
WRKY18
ChIP-seq
EW8
b
Chitin
Pep2
nlp20
INA
EA 1671
Relative Expression
0h 1h 3h
***
c
pGL3B_Enhancer-mini35s::LUC
pause site
Enhancer
mini35S
LUC
AmpR
EA922 WT 142 CCGTTGACTTTGAA TATATTGACTTGGAT 178 bp
w-box CCGTTAAATTTGAA TATATTAAATTGGAT
EA1007 WT 124 GTTTTTTGACTAAAT ATTTTTGACCAAAGA 253 bp
w-box GTTTTTTAAATAAAT ATTTTTAAACAAAGA
EA2747 WT 858 CACTTGACTTATAGTTTACATATAATTGACTTTCC 415 bp
w-box CAC TCC
ED1285 WT 651 AAAAACTAATACAATTTGACTCGTTCTATTTCTTA 218 bp
w-box AAAAACTAATACAATTTAAATCGTTCTATTTCTTA
EW8 WT 464 ACTTGACCT GAATTGACTC GTTGACCCC 66 bp
w-box ACTTAAACT GAA CCC
